# Coulomb Interaction-Controlled Coherence and Entanglement in a Double Quantum Dot Thermoelectric Engine

**DOI:** 10.3390/e28080923

**Published:** 2026-08-18

**Authors:** Rongqian Wang, Le Wang, Zelin Kong, Xiaoping Ma, Yuxin Xu, Ziming Wang, Jia Tan, Xiang Hao, Jincheng Lu

**Affiliations:** 1 School of Digital Low-Altitude, Suzhou Polytechnic University, Suzhou 215104, China; 2 School of Physical Science and Technology, Soochow University, Suzhou 215006, China; 3 Key Laboratory of Intelligent Optoelectronic Devices and Chips of Jiangsu Higher Education Institutions, School of Physical Science and Technology, Suzhou University of Science and Technology, Suzhou 215009, China; 4 Advanced Technology Research Institute of Taihu Photon Center, School of Physical Science and Technology, Suzhou University of Science and Technology, Suzhou 215009, China

**Keywords:** double quantum dot, quantum coherence, heat engine

## Abstract

We study the thermoelectric performance and stationary quantum correlations of a coherent double quantum dot heat engine driven solely by two conventional electronic reservoirs. The role of coherence is isolated by comparing the fully coherent dynamics with those under an energy-conserving pure dephasing channel that does not alter the system energy. Reducing the dephasing strength enhances the particle current, heat current, output power, and thermodynamic efficiency over a broad voltage range. After optimizing the electrochemical load and the dot energy levels, we find that coherence primarily amplifies the attainable power and efficiency without significantly relocating the optimal operating region. Although appreciable interdot coherence already exists at moderate Coulomb interaction, stationary entanglement emerges only when Coulomb blockade sufficiently suppresses the mixed-state contribution from the empty and doubly occupied states. We further construct a transport-based lower bound on the concurrence, providing an experimentally accessible entanglement witness that avoids full state tomography. These findings establish a clear hierarchy among energy filtering, quantum coherence, and Coulomb blockade in a minimal quantum thermoelectric device.

## 1. Introduction

Quantum thermal machines extend the concepts of classical heat engines, refrigerators, and heat pumps to microscopic systems whose dynamics are governed by quantum states, discrete energy spectra, and reservoir-induced transitions [1,2,3,4,5,6,7]. A central question in this field is whether genuinely non-classical resources such as quantum superposition, coherence, and entanglement can improve the performance, stability, or precision of energy conversion processes [8,9,10,11,12,13,14]. Identifying a genuine quantum advantage requires considerable care. Changes in current or power can arise equally well from a modified energy spectrum, altered coupling strengths, or increased thermal occupation [15,16]. A meaningful assessment therefore demands a comparison in which the classical and quantum descriptions share identical energy levels, reservoir parameters, and thermodynamic forces. Furthermore, different forms of quantumness must be distinguished because coherence, entanglement, and purity describe physically distinct properties of a nonequilibrium state [17,18,19,20].

In transport systems containing coherently coupled quantum dots, the interdot particle current is directly proportional to the imaginary part of the local coherence [21,22,23,24,25,26,27]. This relation implies that stationary transport cannot always be described through populations alone. When the coherent energy splitting becomes comparable to the dissipative broadening, a fully secular master equation may artificially suppress the coherences that are essential for transport. A reduced dynamical description that retains relevant nonsecular terms is required in this regime [28,29]. Entanglement represents a more restrictive form of quantum correlation. A state can possess finite coherence without being entangled, while an entangled state necessarily contains correlations that cannot be reproduced by any separable mixture [30,31,32]. In a double quantum dot, the concurrence between the two occupation modes depends on the coherence between the singly occupied states but also on the populations of the empty and doubly occupied states. A finite coherence is therefore necessary but not sufficient for nonzero entanglement [33,34,35,36].

The relation between entanglement and thermodynamic performance remains largely unresolved. A highly entangled state does not necessarily carry a large current, and a large current does not guarantee entanglement. The operating points of maximum power, maximum efficiency, and maximum entanglement generally need not coincide [37,38]. Clarifying the connections among these quantities is essential for determining whether entanglement serves as an active thermodynamic resource or merely accompanies the transport process. A further challenge concerns the experimental identification of stationary entanglement. Full state tomography is impractical in a transport device, motivating the search for relations that connect entanglement to directly measurable quantities. Because the stationary interdot current is proportional to the imaginary part of the local coherence, it can provide a lower bound on the coherence magnitude and, when combined with the empty and doubly occupied probabilities, yields a sufficient transport criterion for nonzero concurrence.

The remainder of this paper is organized as follows. Section 2 presents the double quantum dot model, and the coherence-preserving master equation, the full counting statistics formalism, and the measures of quantum correlations are derived in Section 3. Section 4 analyzes the thermoelectric performance, stationary coherence, concurrence, and current fluctuations, together with the entanglement witness. Section 5 summarizes the main findings and discusses possible extensions.

## 2. Microscopic Transport Framework for an Interacting Double Quantum Dot

**Figure 1:**
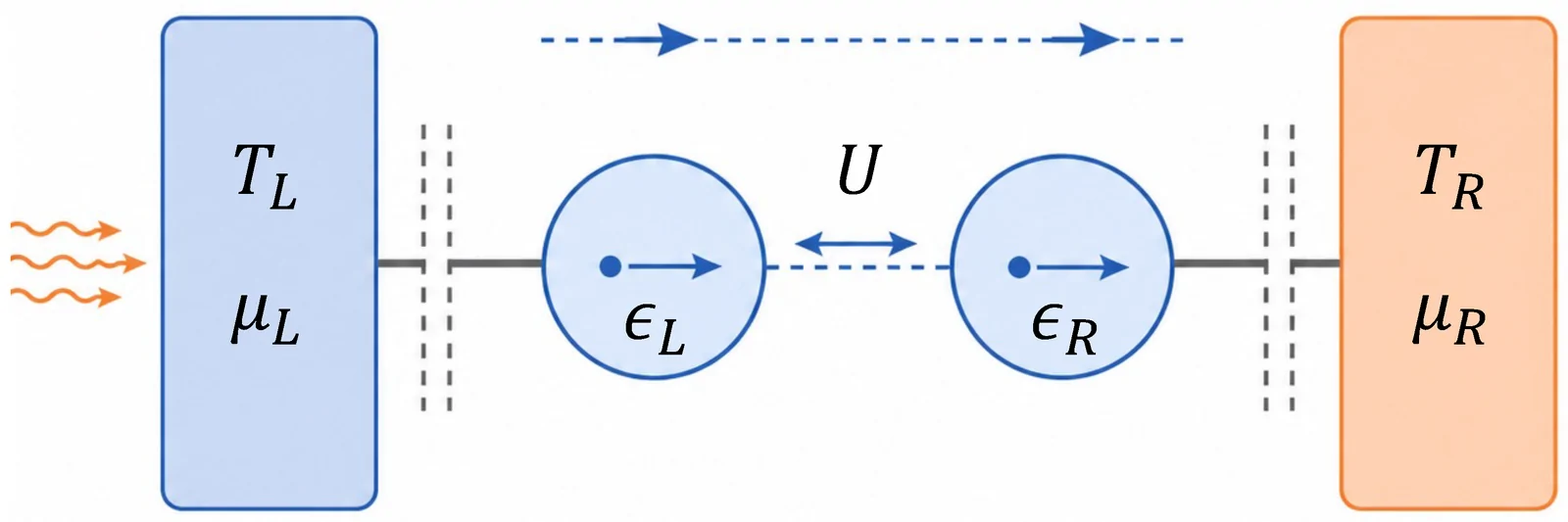
Schematic representation of the coherent double quantum dot thermoelectric heat engine. The left and right quantum dots with localized energies $\epsilon_{L}$ and $\epsilon_{R}$ are coupled through the coherent tunneling amplitude $t_{c}$ and interact through the interdot Coulomb energy *U*. Each quantum dot exchanges electrons with its adjacent electronic reservoir through the coupling strengths $\Gamma_{L}$ and $\Gamma_{R}$. The left reservoir is maintained at a higher temperature than the right reservoir, with $T_{L}>T_{R}$, and supplies heat to the central system. The chemical potentials are chosen such that $\mu_{R}>\mu_{L}$, allowing the temperature difference to drive electrons from the left reservoir to the right reservoir against the electrochemical bias. The solid arrows indicate the direction of particle transport, while the wavy arrows represent the heat absorbed from the hot reservoir. The double-headed arrow between the quantum dots denotes coherent interdot charge transfer. No additional bosonic or nonthermal reservoir is included.

We consider a coherent double quantum dot coupled to two conventional electronic reservoirs with independently controlled temperatures and chemical potentials, as illustrated in Figure 1. The central system consists of two spinless quantum dots connected by a coherent tunneling amplitude and subject to a finite interdot Coulomb interaction. Each quantum dot exchanges electrons only with its adjacent equilibrium reservoir. The temperature difference drives particle and energy transport, while the chemical potential difference acts as an opposing electrochemical load. All nonequilibrium resources therefore originate from the two electronic reservoirs, with no additional driving field or auxiliary environment. This minimal configuration enables us to examine how coherence generated by interdot tunneling and charge population imbalance influences thermoelectric energy conversion and stationary mode entanglement. The total Hamiltonian reads [39,40]

(1) $$H=H_{S}+\sum_{\nu =L,R}H_{\nu }+H_{T},$$

where $H_{S}$ describes the double quantum dot, $H_{\nu }$ the electronic reservoir labeled by ν, and $H_{T}$ the tunneling between the central system and the reservoirs. Throughout this work, we adopt the convention $\hbar =k_{B}=e=1$ unless physical units are restored for clarity.

The spinless double quantum dot is governed by

(2) $$H_{S}=\epsilon_{L}d_{L}^{†}d_{L}+\epsilon_{R}d_{R}^{†}d_{R}+t_{c}\left(d_{L}^{†}d_{R}+d_{R}^{†}d_{L}\right)+Un_{L}n_{R}.$$

Here, $d_{\nu }^{†}$ and $d_{\nu }$ create and annihilate an electron on dot ν, and the corresponding occupation is $n_{\nu }=d_{\nu }^{†}d_{\nu }$. The parameters $\epsilon_{L}$ and $\epsilon_{R}$ are the local site energies, $t_{c}$ denotes the coherent interdot tunneling amplitude, and *U* represents the interdot Coulomb interaction. The spinless description is appropriate when spin degeneracy is lifted by a large magnetic field or when only a single spin channel participates in transport. The model retains the empty state, the two singly occupied states, and the doubly occupied state. The single-level approximation is justified when the orbital level spacing inside each dot exceeds the thermal and electrochemical transport windows, so that higher orbital states remain weakly populated and can be neglected. Under this assumption, two electrons cannot occupy the same local orbital, whereas the state |2〉 remains available because the two electrons reside on different dots. The concurrence discussed later refers to entanglement between the left and right occupation modes rather than to spin entanglement.

The electronic reservoirs are non-interacting fermionic continua described by $H_{\nu }=\sum_{k}\varepsilon_{\nu k}c_{\nu k}^{†}c_{\nu k}$ and $N_{\nu }=\sum_{k}c_{\nu k}^{†}c_{\nu k}$, where $c_{\nu k}^{†}$ creates an electron of energy $\varepsilon_{\nu k}$ in reservoir ν, and the index *k* labels the single-particle modes (e.g., momentum states) of reservoir ν. Each reservoir is initially prepared in the grand canonical state $\rho_{\nu }=Z_{\nu }^{-1}\exp\left[-\beta_{\nu }\left(H_{\nu }-\mu_{\nu }N_{\nu }\right)\right]$, with the inverse temperature $\beta_{\nu }=1/T_{\nu }$ and chemical potential $\mu_{\nu }$.

The tunneling Hamiltonian reads

(3) $$H_{T}=\sum_{\nu =L,R}\sum_{k}\left(V_{\nu k}d_{\nu }^{†}c_{\nu k}+V_{\nu k}^{*}c_{\nu k}^{†}d_{\nu }\right).$$

The left reservoir couples exclusively to the left dot, and the right reservoir to the right dot, establishing a direct connection between interdot coherence and stationary charge transport.

The spinless description is appropriate when spin degeneracy is lifted by a large magnetic field or when only a single spin channel participates in transport. The model retains the empty state, the two singly occupied states, and the doubly occupied state. The local occupation basis is taken as [39,41]

(4) |0〉=|00〉,|L〉=|10〉,|R〉=|01〉,|2〉=|11〉,

and in the absence of coherent tunneling the corresponding energies are $E_{0}=0$, $E_{L}=\epsilon_{L}$, $E_{R}=\epsilon_{R}$, and $E_{2}=\epsilon_{L}+\epsilon_{R}+U$. Finite *U* is retained because it controls the occupation of the doubly occupied state and determines whether the coherence between the singly occupied states qualifies as genuine entanglement between the two occupation modes. The strong Coulomb blockade regime is reached when $U\gg T_{\nu },\Gamma_{\nu },\left|\epsilon_{\nu }-\mu_{\nu }\right|$, where the probability of the state |2〉 becomes strongly suppressed. The coherent interdot tunneling term $t_{c}\left(d_{L}^{†}d_{R}+d_{R}^{†}d_{L}\right)$ in Equation (2) hybridizes the localized states |L〉 and |R〉. Introducing the average energy $\bar{\epsilon }=\left(\epsilon_{L}+\epsilon_{R}\right)/2$ and the level detuning $\delta =\epsilon_{L}-\epsilon_{R}$, the energy splitting of the hybridized states is $\Omega =\sqrt{\delta^{2}+4t_{c}^{2}}$. The mixing angle φ is determined by $\tan\left(2\varphi \right)=2t_{c}/\delta$, and the one-electron eigenstates can therefore be written as [42]

(5) |+〉=cosφ|L〉+sinφ|R〉,|−〉=−sinφ|L〉+cosφ|R〉,

with eigenenergies $E_{\pm }=\bar{\epsilon }\pm \Omega /2$. The mixing angle dictates how strongly each hybridized eigenstate couples to the left and right reservoirs. Close to resonance (δ≈0) the two eigenstates become delocalized over both dots, and coherence between the local states |L〉 and |R〉 can become substantial. For large detuning, the eigenstates approach the localized states, and the role of interdot coherence is correspondingly reduced.

## 3. Quantum Master Equation Approach with Finite Coarse-Graining

### 3.1. Coherence-Preserving Reduced Dynamics

To describe the stationary transport properties of the double quantum dot, we trace out the electronic reservoirs and derive an effective equation of motion for the reduced density matrix $\rho \left(t\right)={\mathrm{Tr}}_{B}\left[\rho_{\mathrm{tot}}\left(t\right)\right]$. Assuming weak system-reservoir coupling, an initially factorized state, and sufficiently short reservoir correlation times, the reduced dynamics can be obtained within the Born–Markov framework.

A central difficulty arises when the energy splitting $\Omega =E_{+}-E_{-}$ of the hybridized one-electron states becomes comparable to the dissipative broadening induced by the reservoirs. The standard secular approximation eliminates coherences between different transition frequencies and may therefore artificially destroy the very interdot coherence that the present work aims to investigate. To avoid this loss, we adopt a finite coarse-graining scheme that retains dissipative contributions involving nearby transition frequencies.

The local annihilation operators are first decomposed into eigenoperators of $H_{S}$

(6) $$d_{\nu }\left(\omega \right)=\sum_{E_{b}-E_{a}=\omega }\Pi_{a}d_{\nu }\Pi_{b},$$

where $\Pi_{a}$ projects onto the eigenspace with energy $E_{a}$ and ω labels the corresponding transition frequency. These operators satisfy $\left[H_{S},d_{\nu }\left(\omega \right)\right]=-\omega d_{\nu }\left(\omega \right)$. Expressing the tunneling Hamiltonian as $H_{T}=\sum_{\nu }\left(d_{\nu }^{†}B_{\nu }+B_{\nu }^{†}d_{\nu }\right)$ with the bath operator $B_{\nu }=\sum_{k}V_{\nu k}c_{\nu k}$, the influence of the reservoirs enters through the correlation functions $C_{\nu }^{\mathrm{in}}\left(s\right)=\langle B_{\nu }^{†}\left(0\right)B_{\nu }\left(s\right)\rangle$ and $C_{\nu }^{\mathrm{out}}\left(s\right)=\langle B_{\nu }\left(s\right)B_{\nu }^{†}\left(0\right)\rangle$.

For a finite coarse-graining time, $\tau_{\mathrm{cg}}$ the dissipative coefficients read

(7) $$\gamma_{\nu ,\tau_{\mathrm{cg}}}^{\mathrm{in}/\mathrm{out}}\left(\omega ,\omega^{\prime }\right)=\frac{1}{\tau_{\mathrm{cg}}}\int_{0}^{\tau_{\mathrm{cg}}}dt_{1}\int_{0}^{\tau_{\mathrm{cg}}}dt_{2}\;e^{i\omega t_{1}}e^{-i\omega^{\prime }t_{2}}C_{\nu }^{\mathrm{in}/\mathrm{out}}\left(t_{1}-t_{2}\right).$$

The coarse-graining time is chosen to exceed the reservoir correlation time while remaining shorter than the characteristic relaxation time of the system. Terms with $\omega \neq \omega^{\prime }$ are kept whenever the corresponding frequency difference is not resolved on the scale set by $\tau_{\mathrm{cg}}$. These terms couple populations and coherences and become essential when Ω is comparable to $\Gamma_{L}$, $\Gamma_{R}$, or the thermal energy [42].

With these ingredients, the reduced master equation takes the form [5,43]

(8) $$\frac{d\rho }{dt}=-i\left[H_{S}+H_{\mathrm{LS}},\rho \right]+\sum_{\nu =L,R}\left(L_{\nu }^{\mathrm{in}}+L_{\nu }^{\mathrm{out}}\right)\rho ,$$

where $H_{\mathrm{LS}}$ is the Lamb shift Hamiltonian. In the weak-coupling regime, this term provides a reservoir-induced renormalization of the system energies and coherent couplings. In the numerical calculations presented below, the Lamb shift is not treated as an independent contribution; its static part is absorbed into the effective values of $\epsilon_{L}$, $\epsilon_{R}$, and $t_{c}$. The injection dissipator is

(9) $$L_{\nu }^{\mathrm{in}}\rho =\sum_{\omega ,\omega^{\prime }}\gamma_{\nu ,\tau_{\mathrm{cg}}}^{\mathrm{in}}\left(\omega ,\omega^{\prime }\right)\left[d_{\nu }^{†}\left(\omega \right)\rho d_{\nu }\left(\omega^{\prime }\right)-\frac{1}{2}\left\{d_{\nu }\left(\omega^{\prime }\right)d_{\nu }^{†}\left(\omega \right),\rho \right\}\right],$$

and the extraction dissipator is obtained by replacing in→out and exchanging the roles of $d_{\nu }$ and $d_{\nu }^{†}$. Each dissipator $L_{\nu }^{\mathrm{in}/\mathrm{out}}$ describes electron injection or extraction from reservoir ν and contains the counting fields required for full counting statistics [44]. The explicit matrix representation of these dissipators in the eigenbasis of $H_{S}$ is provided in Appendix A.

In the limit of very long coarse-graining time, the coefficients become diagonal in the transition frequencies and reduce to the familiar Fermi–Dirac rates $\gamma_{\nu }^{\mathrm{in}}\left(\omega ,\omega \right)=\Gamma_{\nu }f_{\nu }\left(\omega \right)$ and $\gamma_{\nu }^{\mathrm{out}}\left(\omega ,\omega \right)=\Gamma_{\nu }\left[1-f_{\nu }\left(\omega \right)\right]$. This secular limit provides an incoherent reference against which coherent transport effects can be systematically identified. The spectral coupling function $\Gamma_{\nu }\left(\omega \right)=2\pi \sum_{k}{\left|V_{\nu k}\right|}^{2}\delta \left(\omega -\varepsilon_{\nu k}\right)$ characterizes the frequency-dependent coupling to reservoir ν. Within the wide-band approximation, we set $\Gamma_{\nu }\left(\omega \right)≃\Gamma_{\nu }$ over the energy interval relevant for transport, and the electronic occupation is given by the Fermi–Dirac distribution $f_{\nu }\left(\omega \right)={\left\{\exp\left[\beta_{\nu }\left(\omega -\mu_{\nu }\right)\right]+1\right\}}^{-1}$. Differences in temperature and chemical potential provide the thermodynamic forces. A thermoelectric heat engine is realized when a temperature difference ΔT drives electrons against the imposed chemical potential bias Δμ. The stationary density matrix $\rho_{\mathrm{ss}}$ is obtained by solving $L\left(0\right)\rho_{\mathrm{ss}}=0$ with the normalization condition $\mathrm{Tr}(\rho_{\mathrm{ss}})=1$. Numerically, this is done by vectorizing ρ and finding the right eigenvector of the Liouvillian corresponding to the zero eigenvalue [45].

### 3.2. Controlled Dephasing Benchmark

To assess the role of phase coherence without modifying the system Hamiltonian or its energy spectrum, we introduce an energy-conserving pure dephasing channel in the hybridized one-electron eigenbasis. The corresponding Hermitian dephasing operator is defined as

(10) $$A_{\phi }=|+\rangle \;\langle +|-|-\rangle \;\langle -|,$$

where |+〉 and |−〉 are the hybridized one electron eigenstates introduced in Equation (5). The operator $A_{\phi }$ acts trivially on the empty and doubly occupied states.

The dephasing contribution to the master equation is

(11) $$L_{\phi }\rho =\frac{\gamma_{\phi }}{2}D\left[A_{\phi }\right]\rho ,$$

where

(12) $$D\left[A\right]\rho =A\rho A^{†}-\frac{1}{2}\left\{A^{†}A,\rho \right\}$$

is the standard Lindblad dissipator. Since

(13) $$[A_{\phi },H_{S}]=0,$$

the dephasing channel does not exchange energy directly with the double quantum dot. Indeed,

(14) $$J_{\phi }^{E}=\mathrm{Tr}\left[H_{S}L_{\phi }\rho \right]=0.$$

The channel leaves the populations in the energy eigenbasis unchanged and suppresses the coherence between the hybridized states according to

(15) $${\left.\frac{d\rho_{+-}}{dt}\right|}_{\phi }=-\gamma_{\phi }\rho_{+-}.$$

The local interdot coherence $\rho_{LR}$ contains both a contribution from $\rho_{+-}$ and a contribution associated with the populations of the hybridized states. The present channel therefore suppresses the phase-sensitive component of the local coherence without shifting the energy levels or modifying the coherent tunneling amplitude.

The limit $\gamma_{\phi }=0$ corresponds to the coherence-preserving dynamics. Increasing $\gamma_{\phi }$ progressively suppresses the phase coherence between the hybridized one-electron states. The large $\gamma_{\phi }$ regime provides an energy basis dephased reference with the same dot energies, Coulomb interaction, interdot tunneling amplitude, and reservoir parameters. Comparing the coherence-preserving and dephased dynamics therefore identifies the transport contribution associated with phase coherence while excluding changes caused by a modified system spectrum.

### 3.3. Counting Field Formulation of Particle and Energy Transport

To access average currents and their fluctuations, we employ the formalism of full counting statistics. We introduce particle and energy counting fields for each electronic reservoir, collectively denoted by $\chi =\{\chi_{L}^{N},\chi_{L}^{E},\chi_{R}^{N},\chi_{R}^{E}\}$, and define positive currents as particle or energy transfer from a reservoir into the double quantum dot [46]. The counting-field-resolved density matrix ρ(χ,t) then evolves according to [43]

(16) $$\frac{d}{dt}\rho \left(\chi ,t\right)=L\left(\chi \right)\rho \left(\chi ,t\right),$$

where the Liouvillian L(χ) is constructed by inserting appropriate phase factors into the jump operators of the master equation. Specifically, for a transition involving frequencies ω and $\omega^{\prime }$ with the mean transition energy ${\bar{\omega }}_{\omega \omega^{\prime }}=\left(\omega +\omega^{\prime }\right)/2$, the injection term corresponding to reservoir ν is multiplied by $\exp(i\chi_{\nu }^{N}+i\chi_{\nu }^{E}{\bar{\omega }}_{\omega \omega^{\prime }})$, while the extraction term is multiplied by $\exp(-i\chi_{\nu }^{N}-i\chi_{\nu }^{E}{\bar{\omega }}_{\omega \omega^{\prime }})$. The anticommutator contributions remain unchanged because they do not correspond to detected transfer events.

The moment-generating function Z(χ,t)=Tr[ρ(χ,t)] encodes the cumulants of the transferred quantities. Its long-time limit defines the scaled cumulant generating function [47]

(17) $$G\left(\chi \right)=\lim_{t\to \infty }\frac{1}{t}\ln Z\left(\chi ,t\right),$$

which satisfies G(0)=0. The stationary particle and energy currents from reservoir ν into the system follow from the first derivatives [48,49]

(18) $$\begin{array}{cc} J_{\nu }^{N} & ={\left.\frac{\partial G}{\partial (i\chi_{\nu }^{N})}\right|}_{\chi =0}, \end{array}$$

(19) $$\begin{array}{cc} J_{\nu }^{E} & ={\left.\frac{\partial G}{\partial (i\chi_{\nu }^{E})}\right|}_{\chi =0}. \end{array}$$

In the stationary state particle and energy conservation impose $J_{L}^{N}+J_{R}^{N}=0$ and $J_{L}^{E}+J_{R}^{E}=0$.

### 3.4. Thermodynamic Observables

The heat current entering the double quantum dot from reservoir ν is defined as [50]

(20) $$J_{\nu }^{Q}=J_{\nu }^{E}-\mu_{\nu }J_{\nu }^{N},$$

which separates the energy carried by particles from the chemical work associated with particle exchange. At the stationary state, the system entropy remains constant, and the entropy production rate takes the compact form ${\dot{\Sigma }}_{\mathrm{ss}}=-\sum_{\nu =L,R}\frac{J_{\nu }^{Q}}{T_{\nu }}\geq 0$, consistent with the second law of thermodynamics [51].

The electrical power delivered to an external load can be written, using particle conservation, as [52,53,54]

(21) $$P_{\mathrm{out}}=\left(\mu_{R}-\mu_{L}\right)J_{L}^{N}.$$

The device operates as a heat engine when $P_{\mathrm{out}}>0$ and heat is extracted from the hotter reservoir. Denoting by *h* the reservoir with the higher temperature, the thermoelectric efficiency is [54,55]

(22) $$\eta =\frac{P_{\mathrm{out}}}{J_{h}^{Q}},$$

which is bounded from above by the Carnot efficiency $\eta_{C}=1-T_{c}/T_{h}$ with $T_{c}$ and $T_{h}$ the temperatures of the cold and hot reservoirs, respectively. The efficiency at maximum power is obtained by maximizing $P_{\mathrm{out}}$ over the chemical potential difference Δμ, yielding the characteristic quantities $P_{\max}=\max_{\Delta \mu }P_{\mathrm{out}}$ and $\eta_{\mathrm{MP}}{=\eta |}_{P_{\mathrm{out}}=P_{\max}}$. These observables allow the interplay between quantum correlations and thermodynamic performance to be examined under identical external conditions [56,57].

### 3.5. Quantum Coherence and Occupation Mode Entanglement

The local occupation basis introduced in Equation (4) provides a natural description of both coherence and quantum correlations in the double quantum dot. The four many-body states can be written as

(23) $$|0\rangle =|0_{L}0_{R}\rangle ,|L\rangle =|1_{L}0_{R}\rangle ,|R\rangle =|0_{L}1_{R}\rangle ,|2\rangle =|1_{L}1_{R}\rangle ,$$

where the two entries label the occupations of the left and right local fermionic modes.

The stationary density matrix inherits the charge-sector structure of the reduced dynamics. The system Hamiltonian commutes with the total particle number operator $N_{S}=n_{L}+n_{R}$. Although the electronic reservoirs exchange particles with the double quantum dot, they are ordinary equilibrium reservoirs without a coherent phase reference, and the resulting Liouvillian is covariant under global U(1) transformations generated by $N_{S}$. For a unique stationary state, this implies $[N_{S},\rho_{\mathrm{ss}}]=0$, so that coherences between different charge sectors vanish identically. In particular, $\rho_{0L}=\rho_{0R}=\rho_{02}=\rho_{L2}=\rho_{R2}=0$. The empty and doubly occupied sectors are one-dimensional and therefore trivially diagonal. The only sector with dimension larger than one is the singly occupied sector spanned by |L〉 and |R〉. Within this sector, coherent interdot tunneling $H_{\mathrm{tun}}=t_{c}\left(|L\rangle \;\langle R|+|R\rangle \;\langle L|\right)$ couples the two basis states and can maintain the off-diagonal element $\rho_{LR}=\langle L|\rho_{\mathrm{ss}}|R\rangle$. No analogous Hamiltonian term couples state belonging to different charge sectors, and the reservoir-induced injection and extraction processes are purely incoherent. Hence $\rho_{LR}$ and its Hermitian conjugate are the only off-diagonal elements that can be non-zero in the stationary state, and the density matrix takes the *X*-state form

(24) $$\rho_{\mathrm{ss}}=\left(\begin{array}{cccc} p_{0} & 0 & 0 & 0\\ 0 & p_{L} & \rho_{LR} & 0\\ 0 & \rho_{LR}^{*} & p_{R} & 0\\ 0 & 0 & 0 & p_{2} \end{array}\right),$$

with $p_{0}+p_{L}+p_{R}+p_{2}=1$. The elements $\rho_{LR}$ and $\rho_{LR}^{*}$ are the only stationary off-diagonal entries allowed by charge-sector conservation and coherent interdot coupling. The pure dephasing channel preserves this block structure; it can suppress $\rho_{LR}$ but cannot create coherences between different particle number sectors. The $l_{1}$-norm of coherence, defined as the sum of the absolute values of all off-diagonal elements, reduces for this state to $C_{l_{1}}=2\left|\rho_{LR}\right|$. This quantity measures coherence in the local occupation basis and directly reflects delocalization between the left and right dots.

The entanglement considered here is the fermionic mode entanglement between the left and right occupation modes. It does not refer to spin entanglement, because spin degrees of freedom are absent, nor to entanglement between two distinguishable electrons. Each spinless orbital spans a two-dimensional Fock space $H_{\alpha }=\mathrm{span}\left\{|0_{\alpha }\rangle ,|1_{\alpha }\rangle \right\}$ for α=L,R. After fixing the mode ordering, the occupation state in Equation (24) has the algebraic structure of a two-qubit *X*-state. The Wootters concurrence can therefore be evaluated for the left-right occupation-mode partition [58,59,60] and reduces exactly to

(25) $$C_{\mathrm{mode}}=2\max\;\left\{0,\;|\rho_{LR}|-\sqrt{p_{0}p_{2}}\right\}.$$

We refer to $C_{\mathrm{mode}}$ as the occupation-mode concurrence.

Equation (25) also clarifies why finite coherence does not guarantee entanglement. The coherent contribution $|\rho_{LR}|$ must exceed the mixed-occupation background $\sqrt{p_{0}p_{2}}$, which accounts for the incoherent admixture of the empty and doubly occupied configurations. Increasing the Coulomb interaction suppresses $p_{2}$ and thereby lowers the threshold for nonzero mode concurrence, even without enhancing the coherence itself. In the strong Coulomb blockade limit where $p_{2}\to 0$, one obtains $C_{\mathrm{mode}}≃2\left|\rho_{LR}\right|$.

The connection between coherence and transport is provided by the interdot particle current operator. In units with ℏ=1, ${\hat{J}}_{LR}=it_{c}\left(d_{L}^{†}d_{R}-d_{R}^{†}d_{L}\right)$, and its stationary expectation value is

(26) JLR=TrJ^LRρss=2tcImρLR.

Restoring physical units, the electric current reads ILR=(2etc/ℏ)ImρLR. Hence, the stationary current probes only the imaginary part of the interdot coherence.

Since |ρLR|≥|ImρLR|=ℏ|ILR|/(2e|tc|), the mode concurrence satisfies the lower bound

(27) $$C_{\mathrm{mode}}\geq W_{I},\;W_{I}=2\max\;\left\{0,\;\frac{\hbar |I_{LR}|}{2e|t_{c}|}-\sqrt{p_{0}p_{2}}\right\}.$$

A positive $W_{I}$ certifies nonzero occupation-mode concurrence using only the stationary interdot current and the probabilities of the empty and doubly occupied configurations, the latter being accessible through charge-sensing measurements. The bound becomes tight when the stationary coherence is predominantly imaginary. A finite real part of $\rho_{LR}$ contributes to $C_{\mathrm{mode}}$ without affecting the current, so a vanishing witness does not imply a separable state. The current witness therefore provides a sufficient condition for entanglement, not a necessary one.

## 4. Thermoelectric Enhancement by Coherence and Entanglement Generation by Coulomb Blockade

This section presents the central numerical results of the paper, organized as follows. Section 4.1 examines how the dephasing strength affects the thermoelectric response at a fixed representative set of quantum dot energies, establishing that quantum coherence enhances the output power, particle current, heat current, and efficiency over a broad voltage range. Section 4.2 extends this analysis to the full energy level plane by independently optimizing the electrochemical load at every point, demonstrating that the coherence advantage persists after optimization and that energy filtering determines the optimal operating region Section 4.3 then addresses the complementary question of stationary entanglement. It shows that the interdot Coulomb interaction *U* controls the conversion of existing coherence into occupation-mode entanglement and that a transport-based witness can certify this entanglement without full state tomography.

### 4.1. Voltage Dependent Thermoelectric Performance

**Figure 2:**
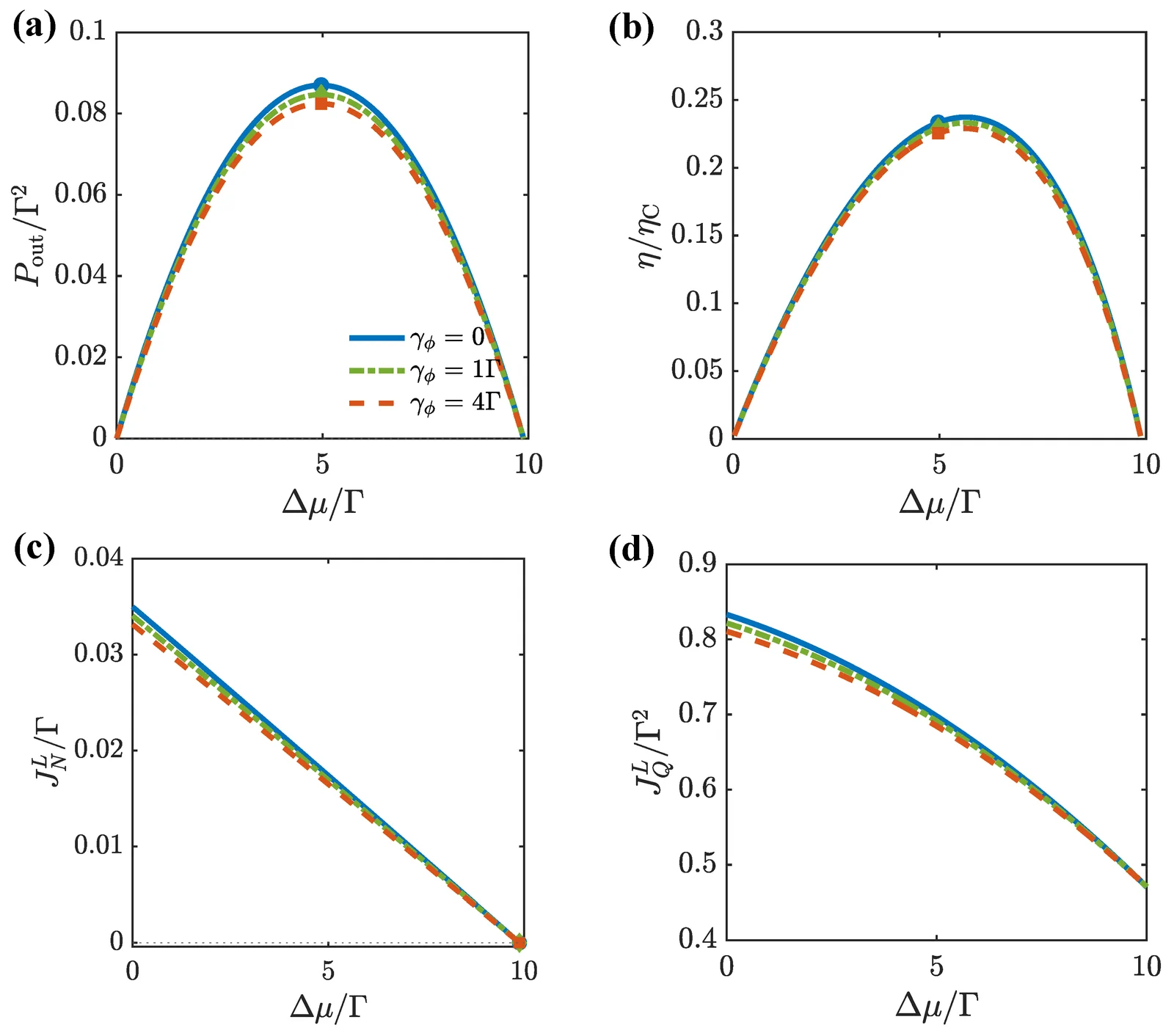
Voltage dependence of the thermodynamic performance of the coherent double quantum dot heat engine for three dephasing strengths. Panels (**a**–**d**) show the output power $P_{\mathrm{out}}$, the normalized efficiency $\eta /\eta_{C}$, the left particle current $J_{N}^{L}$, and the left heat current $J_{Q}^{L}$ as functions of the chemical potential difference Δμ. The blue solid, green dash-dotted, and red dashed curves correspond to $\gamma_{\phi }=0$, $\gamma_{\phi }=\Gamma$, and $\gamma_{\phi }=4\Gamma$, respectively, where finite $\gamma_{\phi }$ progressively suppresses the interdot coherence while conserving the system energy. The symbols in panels (**a**,**b**) mark the maximum power operating points, and those in panel (**c**) mark the stopping condition $J_{N}^{L}=0$. The horizontal dotted line in panel (**b**) indicates the Carnot efficiency. Increasing the dephasing strength reduces both the current and the output power, confirming that stationary quantum coherence supports thermoelectric conversion. Parameters: $\epsilon_{L}=\epsilon_{R}=10\Gamma$, $t_{c}=2\Gamma$, U=160Γ, $\Gamma_{L}=\Gamma_{R}=\Gamma$, $T_{L}=30\Gamma$, $T_{R}=14\Gamma$, $\bar{\mu }=0$.

**Figure 3:**
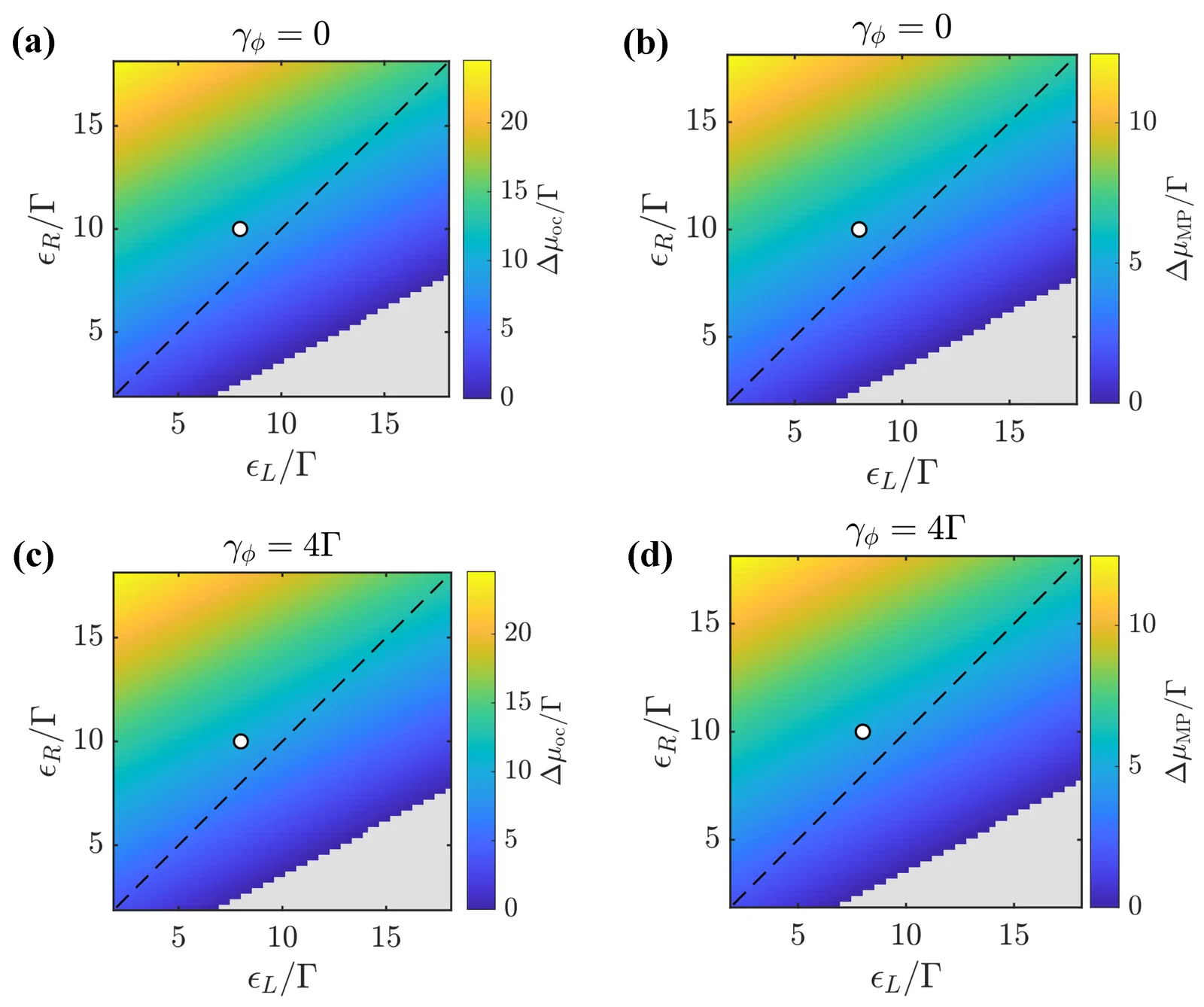
Optimized thermoelectric voltages in the quantum dot energy level plane. (**a**–**d**) show the nonlinear open-circuit voltage $\Delta \mu_{\mathrm{oc}}$ and the voltage at maximum power $\Delta \mu_{\mathrm{MP}}$, respectively. (**a**,**b**) corresponds to the coherence-preserving dynamics with $\gamma_{\phi }=0$, while (**c**,**d**) corresponds to the dephased dynamics with $\gamma_{\phi }=4\Gamma$. For every pair $\left(\epsilon_{L},\epsilon_{R}\right)$, the complete nonlinear current response is calculated as a function of the electrochemical load $\Delta \mu =\mu_{R}-\mu_{L}$. The open-circuit voltage is determined by the condition $J_{N}^{L}\left(\Delta \mu_{\mathrm{oc}}\right)=0$, whereas $\Delta \mu_{\mathrm{MP}}$ is obtained by maximizing $P_{\mathrm{out}}=\Delta \mu J_{N}^{L}$ within the valid heat engine regime satisfying $P_{\mathrm{out}}>0$, $J_{Q}^{L}>0$, and $\dot{\Sigma }\geq 0$. The gray regions denote parameter combinations for which no valid heat engine operating point is found within the considered voltage interval. The dashed line indicates $\epsilon_{L}=\epsilon_{R}$. The remaining parameters are $t_{c}=2\Gamma$, U=80Γ, $\Gamma_{L}=\Gamma_{R}=\Gamma$, $T_{L}=30\Gamma$, $T_{R}=14\Gamma$, and $\bar{\mu }=0$.

We first examine how pure dephasing influences the thermoelectric performance of the double quantum dot heat engine. The parameters used in Figure 2 and Figure 3 are chosen to satisfy the following hierarchy. The dot-reservoir couplings satisfy $\Gamma \ll T_{L},T_{R}$ and Γ≪U, consistent with the weak-coupling Markovian treatment. The interdot tunneling $t_{c}=2\Gamma$ is comparable to the reservoir-induced broadening, ensuring that coherent hybridization remains observable. The dot energies $\epsilon_{L}=8\Gamma$ and $\epsilon_{R}=10\Gamma$ lie above the average chemical potential but within the thermal transport window of the hot reservoir, establishing the energy-filtering condition required for thermoelectric operation. The finite Coulomb interaction U=80Γ provides strong Coulomb blockade while retaining the doubly occupied state in the Hilbert space, which is essential for studying the threshold for occupation-mode entanglement. The temperatures $T_{L}=30\Gamma$ and $T_{R}=14\Gamma$ generate a pronounced nonlinear thermal bias well beyond the linear-response regime. The average chemical potential $\bar{\mu }=0$ merely fixes the reference energy; the load Δμ is optimized independently for every pair of dot energies. Taking Γ=10μeV as an illustrative scale, these parameters correspond to $\epsilon_{L}=80\;\mu \mathrm{eV}$, $\epsilon_{R}=100\;\mu \mathrm{eV}$, $t_{c}=20\;\mu \mathrm{eV}$, U=0.8meV, $k_{B}T_{L}=0.30\;\mathrm{meV}$, and $k_{B}T_{R}=0.14\;\mathrm{meV}$, demonstrating that the dimensionless ratios used in the calculations correspond to realistic mesoscopic energy scales.

Figure 2 presents the output power, efficiency, particle current, and heat current as functions of the chemical potential difference $\Delta \mu =\mu_{R}-\mu_{L}$. The left reservoir is kept at a higher temperature than the right reservoir, with $\mu_{R}>\mu_{L}$, so that the temperature difference drives electrons from left to right against the electrochemical bias. The device operates as a heat engine when $J_{N}^{L}>0$, $J_{Q}^{L}>0$, and $P_{\mathrm{out}}>0$ [61]. Three dephasing strengths are compared. The value $\gamma_{\phi }=0$ corresponds to coherence-preserving dynamics, while $\gamma_{\phi }=\Gamma$ and $\gamma_{\phi }=4\Gamma$ describe intermediate and strong suppression of the coherence between the hybridized one-electron states. All Hamiltonian parameters, reservoir temperatures, chemical potentials, and tunneling rates are kept identical, isolating the influence of phase coherence on the stationary thermoelectric response.

The output power $P_{\mathrm{out}}=\Delta \mu J_{N}^{L}$ shown in Figure 2a rises from zero at Δμ=0, reaches a maximum, and then decreases to zero at the stopping condition. A clear ordering is observed throughout the finite power region. The coherence-preserving case produces the largest output power, and increasing $\gamma_{\phi }$ progressively reduces it, with $P_{\max}^{\gamma_{\phi }=0}>P_{\max}^{\gamma_{\phi }=\Gamma }>P_{\max}^{\gamma_{\phi }=4\Gamma }$. This ordering demonstrates that stationary quantum coherence supports the finite-rate conversion of thermal energy into electrical work. The microscopic origin of this behavior follows from the particle current in Figure 2c. At a fixed bias, the current obeys $J_{N}^{L}{|}_{\gamma_{\phi }=0}>J_{N}^{L}{{|}_{\gamma_{\phi }=\Gamma }>J_{N}^{L}|}_{\gamma_{\phi }=4\Gamma }$ throughout the engine regime. The interdot current operator J^LR=2tcImρLR directly links transport to the local coherence $\rho_{LR}$. Pure dephasing weakens the imaginary part of $\rho_{LR}$ and thereby suppresses the stationary particle current, because every electron transferred between the reservoirs must pass through the interdot junction.

The heat current extracted from the hot reservoir (Figure 2d) remains positive within the engine region and also decreases as $\gamma_{\phi }$ grows. Reducing $\gamma_{\phi }$ therefore enhances the absorbed heat together with the particle current, reflecting the fact that coherent interdot transport increases the rate at which both particles and energy flow through the central system. The efficiency $\eta =P_{\mathrm{out}}/J_{Q}^{L}$ also improves when dephasing is weakened, as shown in Figure 2b. Coherence-preserving dynamics produces a larger electrical output without requiring a proportional increase in the absorbed heat, enhancing both the rate and the quality of thermoelectric energy conversion. The efficiency remains below the Carnot value $\eta_{C}=1-T_{R}/T_{L}$, consistent with the second law. Near the stopping condition, the efficiency can approach a large fraction of $\eta_{C}$ while the power tends to zero, so the efficiency at maximum power is a more meaningful figure of merit for finite-power operation than the largest achievable efficiency. The dephasing channel employed here does not act as an additional energy source. Because $[A_{\phi },H_{S}]=0$, the energy current contributed by the dephasing environment vanishes, $J_{\phi }^{E}=\mathrm{Tr}\left[H_{S}L_{\phi }\rho \right]=0$. The observed changes in power, efficiency, and currents therefore originate solely from the suppression of the phase coherence that supports interdot charge transfer.

In summary, Figure 2 provides direct numerical evidence that internally generated quantum coherence enhances the thermoelectric operation of the double quantum dot heat engine. Weaker dephasing leads to larger particle and heat currents, higher output power, and improved efficiency. The following analysis examines whether this coherence advantage persists after optimization over the load voltage and over the quantum dot energy levels.

### 4.2. Optimized Thermoelectric Performance in the Energy Level Plane

**Figure 4:**
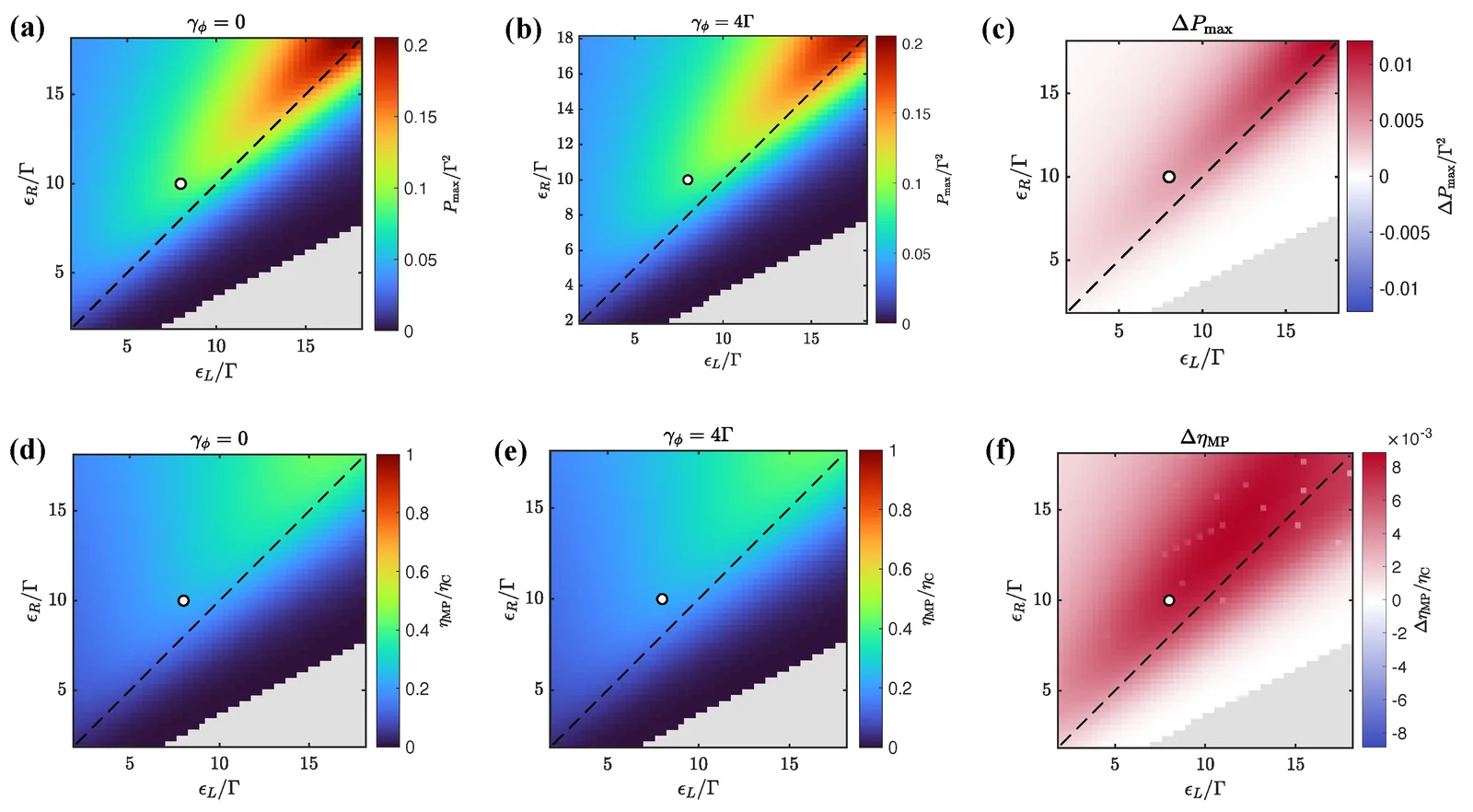
Optimized thermoelectric performance in the energy level plane. Panels (**a**,**b**) show the maximum output power $P_{\max}$ for the coherence-preserving case $\gamma_{\phi }=0$ and the strongly dephased case $\gamma_{\phi }=4\Gamma$, respectively. Panel (**c**) displays their difference $\Delta P_{\max}=P_{\max}\left(\gamma_{\phi }=0\right)-P_{\max}\left(\gamma_{\phi }=4\Gamma \right)$. Panels (**d**,**e**) show the corresponding normalized efficiency $\eta_{\mathrm{MP}}/\eta_{C}$ at maximum power, and panel (**f**) the efficiency difference $\Delta \eta_{\mathrm{MP}}/\eta_{C}$. At each point in the $\left(\epsilon_{L},\epsilon_{R}\right)$ plane, the chemical potential difference Δμ is optimized to deliver the maximal output power, and the efficiency is evaluated at that optimal bias. The diagonal dashed line marks the resonance condition $\epsilon_{L}=\epsilon_{R}$, and the white circle indicates the parameter set used in Figure 2. Positive values in (**c**,**f**) highlight the regions where quantum coherence enhances the thermoelectric performance. The remaining parameters are $t_{c}=2\Gamma$, U=80Γ, $\Gamma_{L}=\Gamma_{R}=\Gamma$, $T_{L}=30\Gamma$, $T_{R}=14\Gamma$, and $\bar{\mu }=0$.

The voltage-dependent results in Figure 2 demonstrate that reducing the dephasing strength enhances the thermoelectric response for a representative choice of quantum dot energies. Figure 4 investigates whether this enhancement persists over a broader parameter range by presenting the maximum output power and the efficiency at maximum power in the $\left(\epsilon_{L},\epsilon_{R}\right)$ plane.

To make the voltage optimization explicit, we also calculate the nonlinear open-circuit voltage and the voltage corresponding to the maximum power point. For each pair $\left(\epsilon_{L},\epsilon_{R}\right)$, the open-circuit voltage $\Delta \mu_{\mathrm{oc}}$ is determined from

(28) $$J_{N}^{L}\left(\Delta \mu_{\mathrm{oc}}\right)=0.$$

At this voltage, the thermally driven current is completely balanced by the opposing electrochemical load. The output power therefore vanishes, and $\Delta \mu_{\mathrm{oc}}$ characterizes the maximum load sustained by the temperature difference rather than a finite power operating point.

For each pair of energy levels, the chemical potential difference is varied independently, and the maximum positive output power within the heat engine regime is selected,

(29) $$P_{\max}\left(\epsilon_{L},\epsilon_{R}\right)=\max_{\Delta \mu \in E}P_{\mathrm{out}}\left(\epsilon_{L},\epsilon_{R},\Delta \mu \right),$$

where $E=\left\{\Delta \mu \;|\;P_{\mathrm{out}}>0,\;J_{Q}^{L}>0,\;\dot{\Sigma }\geq 0\right\}$ denotes the valid heat engine operating regime. The corresponding optimal electrochemical load is denoted by

(30) $$\Delta \mu_{\mathrm{MP}}\left(\epsilon_{L},\epsilon_{R}\right)=\underset{\Delta \mu \in E}{arg max}P_{\mathrm{out}}\left(\epsilon_{L},\epsilon_{R},\Delta \mu \right).$$

Unlike the open-circuit voltage, $\Delta \mu_{\mathrm{MP}}$ represents a finite current operating point. It lies inside the heat engine interval and is therefore smaller than $\Delta \mu_{\mathrm{oc}}$ whenever a regular maximum power point exists.

The efficiency is evaluated at the same optimal load,

(31) $$\eta_{\mathrm{MP}}\left(\epsilon_{L},\epsilon_{R}\right)=\eta \left[\epsilon_{L},\epsilon_{R},\Delta \mu_{\mathrm{MP}}\left(\epsilon_{L},\epsilon_{R}\right)\right].$$

This procedure ensures that the coherent and dephased devices are compared at their respective optimal operating points rather than at an externally fixed bias.

Figure 3 shows the resulting $\Delta \mu_{\mathrm{oc}}$ and $\Delta \mu_{\mathrm{MP}}$ over the quantum dot energy level plane for the coherence-preserving and dephased dynamics [62,63,64]. Comparing these voltage maps with $P_{\max}$ and $\eta_{\mathrm{MP}}$ in Figure 4 distinguishes changes in the optimal load from changes in the thermoelectric performance attained at that load. Interestingly, the optimized voltages exhibit only weak dependence on the dephasing strength. Both $\Delta \mu_{\mathrm{oc}}$ and $\Delta \mu_{\mathrm{MP}}$ remain almost unchanged when increasing $\gamma_{\phi }$ from 0 to 4Γ. This indicates that dephasing does not substantially modify the optimal electrochemical load, but rather reduces the magnitude of the transport response through the suppression of coherent interdot dynamics at the same operating condition. Physically, the pure dephasing channel suppresses the interdot coherence $\rho_{LR}$ and consequently decreases the particle and heat currents, while the voltage position is determined by the zero-current condition $J_{N}\left(\Delta \mu_{\mathrm{oc}}\right)=0$, reflecting the balance between the thermal driving and the electrochemical bias. Consequently, the present results indicate that coherence mainly enhances the attainable thermoelectric performance rather than shifting the location of the optimal operating voltage.

Figure 4a,b show the optimized output power for $\gamma_{\phi }=0$ and $\gamma_{\phi }=4\Gamma$, respectively. In both cases, the largest power is concentrated in the upper part of the scanned energy window, primarily where both $\epsilon_{L}$ and $\epsilon_{R}$ exceed approximately 10Γ. The similar location of the optimal regions indicates that dephasing does not substantially shift the energy level configuration required for efficient heat engine operation. The overall structure of the performance map is determined by energy filtering, thermal occupations, and the alignment of the dot levels with the reservoirs, while quantum coherence mainly modifies the magnitude of the resulting transport. The preference for high quantum dot energies follows from the thermoelectric energy filtering mechanism. The hot left reservoir thermally populates electronic states above the average chemical potential, whereas the colder right reservoir provides a smaller backward occupation. Electrons transmitted through these states carry a comparatively large amount of energy, part of which can be converted into chemical work. The influence of coherence is displayed directly in Figure 4c through the difference $\Delta P_{\max}=P_{\max}^{\gamma_{\phi }=0}-P_{\max}^{\gamma_{\phi }=4\Gamma }$. The largest positive power difference also appears where both dot energies exceed approximately 10Γ, reaching $\Delta P_{\max}≃0.01\Gamma^{2}$. Quantum coherence therefore produces a measurable increase in the optimized output power within the same energy region that already supports efficient thermoelectric conversion. The fact that the high power region remains nearly unchanged while its magnitude increases provides an important interpretation. Coherence does not create an entirely new operating window but reinforces the transport channels selected by the energy level alignment, maintaining a finite phase relation between the localized electronic states and thereby increasing the stationary particle transfer through the interdot junction.

Figure 4d,e show the Carnot-normalized efficiency at maximum power, $\eta_{\mathrm{MP}}/\eta_{C}$, with $\eta_{C}=1-T_{R}/T_{L}$, for the coherence-preserving and strongly dephased dynamics. The regions of large efficiency are again located at relatively high values of both dot energies, and the principal structure remains largely unchanged when the dephasing strength is varied. The corresponding efficiency difference, $\Delta \eta_{\mathrm{MP}}=\left[\eta_{\mathrm{MP}}^{\gamma_{\phi }=0}-\eta_{\mathrm{MP}}^{\gamma_{\phi }=4\Gamma }\right]/\eta_{C}$, is shown in Figure 4f. The largest enhancement reaches approximately $8\times 10^{-3}$, demonstrating that coherence improves not only the maximum output power but also the conversion efficiency at the maximum power point. The efficiency enhancement is smaller in absolute magnitude than the power enhancement because coherence increases both the electrical power and the absorbed heat current. Since $\eta_{\mathrm{MP}}=P_{\max}/J_{Q}^{L}\left(\Delta \mu_{\mathrm{MP}}\right)$, part of the numerator increase is compensated by the simultaneous increase in the denominator. The more pronounced effect on power therefore indicates that the principal role of coherence is to enhance the rate of thermoelectric conversion, while its influence on the conversion ratio remains moderate.

A gray region appears in the lower-right part of the energy-level plane, where $\epsilon_{L}$ is large, while $\epsilon_{R}$ is comparatively small. This region denotes energy level configurations for which no value of the scanned chemical potential difference satisfies the heat engine conditions $P_{\mathrm{out}}>0$, $J_{Q}^{L}>0$, and $\dot{\Sigma }\geq 0$. The excluded points should therefore be interpreted as a nonengine operating region rather than a region of zero optimized power. In this strongly asymmetric configuration, a large $\epsilon_{L}$ suppresses injection from the left reservoir while a low $\epsilon_{R}$ favors occupation from the right, reversing the particle current or preventing transport against the imposed bias. The device may consequently operate as a passive conductor, a dissipative transport channel, or another type of thermal machine, regimes not included in the present heat engine optimization [25,65].

Overall, Figure 4 demonstrates that the thermoelectric operating window is governed predominantly by the quantum dot energy alignment, whereas coherence controls the magnitude of the attainable performance within this window. The coherent and strongly dephased models reach their optimal performance in nearly the same parameter region, but the coherence-preserving device produces both a larger maximum power and a higher efficiency at maximum power. Coherence therefore enhances the performance of an existing thermoelectric transport channel rather than merely shifting the optimal energy levels.

### 4.3. Generation and Transport Signatures of Stationary Entanglement

Quantum coherence is an essential ingredient for quantum transport, but its presence does not guarantee entanglement [12]. In the double quantum dot system, the interdot coherence competes with the incoherent occupation of the empty and doubly occupied states. To distinguish these two effects, we examine the coherence and entanglement properties at the maximum power point as functions of the interdot Coulomb interaction *U*.

**Figure 5:**
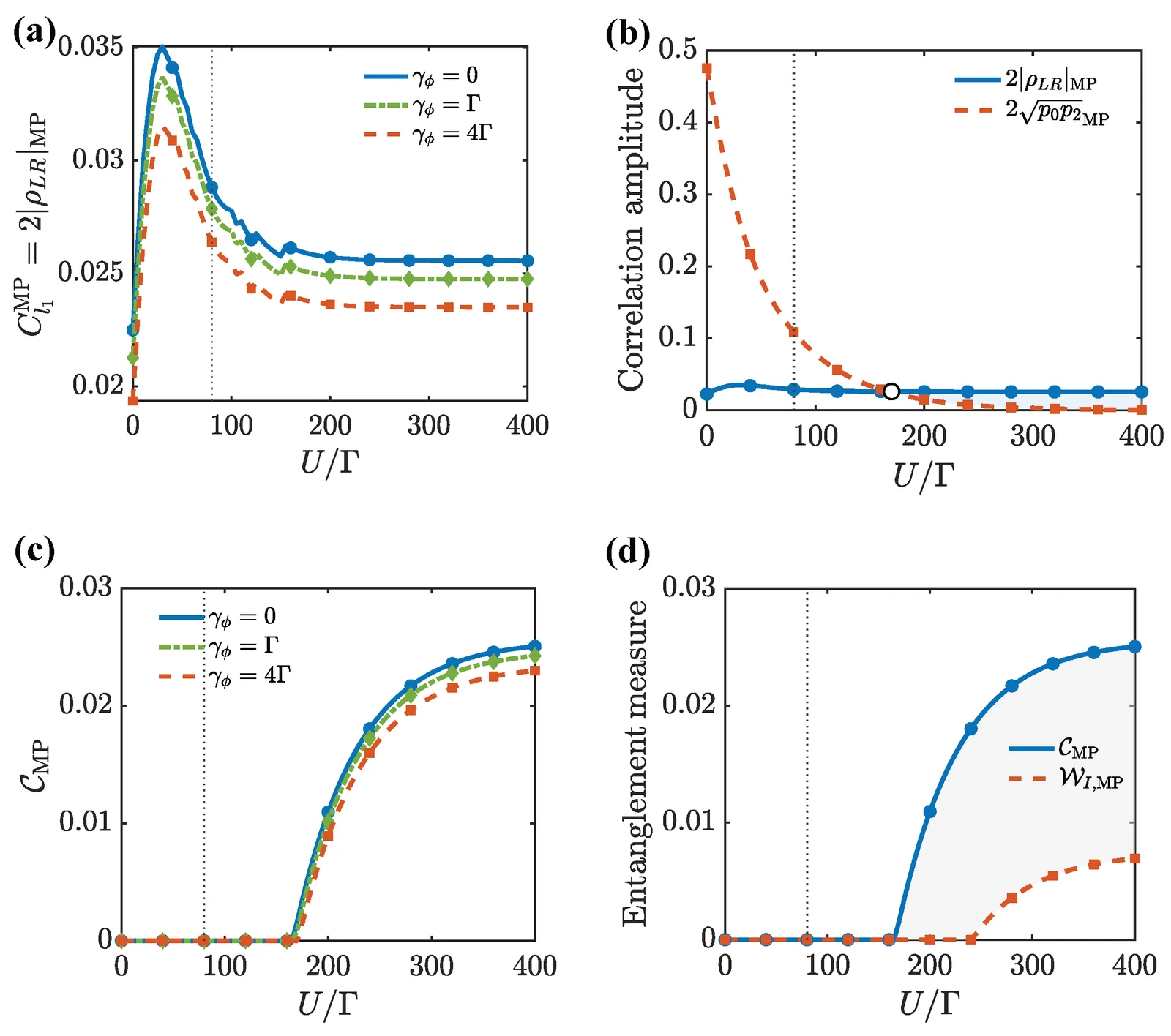
Coulomb blockade-controlled conversion of local coherence into stationary entanglement at the maximum power point. Panel (**a**) shows the local coherence $C_{l_{1}}^{\mathrm{MP}}=2{\left|\rho_{LR}\right|}_{\mathrm{MP}}$ as a function of the interdot Coulomb interaction *U* for three dephasing strengths $\gamma_{\phi }=0$, $\gamma_{\phi }=\Gamma$, and $\gamma_{\phi }=4\Gamma$. Panel (**b**) compares the coherence amplitude $2|\rho_{LR}{|}_{\mathrm{MP}}$ with the entanglement threshold $2{\sqrt{p_{0}p_{2}}}_{\mathrm{MP}}$ for the coherence-preserving case $\gamma_{\phi }=0$, where the blue shaded region marks the regime in which the coherence exceeds the threshold and the stationary state is entangled. The solid circle indicates the crossing point where entanglement first appears with increasing *U*. Panel (**c**) displays the concurrence $C_{\mathrm{MP}}$ as a function of *U* for the three dephasing strengths. Panel (**d**) compares the exact concurrence $C_{\mathrm{MP}}$ with the current-based entanglement witness $W_{I,\mathrm{MP}}$ for $\gamma_{\phi }=0$, illustrating that a positive witness value provides a sufficient experimental criterion for certifying stationary entanglement. All quantities are evaluated at the chemical potential difference Δμ that maximizes the output power at each value of *U*. The vertical dotted line marks the reference interaction strength U=80Γ used in Figure 2 and Figure 3, and the white circle in those figures indicates the fixed energy levels employed here. The remaining parameters are $\epsilon_{L}=8\Gamma$, $\epsilon_{R}=10\Gamma$, $t_{c}=2\Gamma$, $\Gamma_{L}=\Gamma_{R}=\Gamma$, $T_{L}=30\Gamma$, $T_{R}=14\Gamma$, and $\bar{\mu }=0$.

Figure 5a shows the local coherence $C_{l_{1}}^{\mathrm{MP}}=2{\left|\rho_{LR}\right|}_{\mathrm{MP}}$ evaluated at the maximum power point for each value of *U*. At weak Coulomb interaction, the coherence increases rapidly with *U* and reaches a maximum of approximately 0.035, because moderate repulsion suppresses charge fluctuations associated with the doubly occupied state and thereby strengthens the effective single-electron transport channel. For U≳150Γ, the coherence gradually decreases toward a saturated value of about 0.025, indicating that once double occupation is sufficiently suppressed, the system enters a regime dominated by the single-electron subspace. Throughout the entire *U* range, the coherence-preserving case $\gamma_{\phi }=0$ exhibits the largest coherence, followed by $\gamma_{\phi }=\Gamma$ and $\gamma_{\phi }=4\Gamma$, directly confirming that pure dephasing suppresses the stationary phase relation between the two dots while leaving the overall *U* dependence qualitatively unchanged. The relation between coherence and entanglement is examined in Figure 5b. For the present system, the concurrence takes the form $C=2\max\{0,|\rho_{LR}|-\sqrt{p_{0}p_{2}}\}$, where $p_{0}$ and $p_{2}$ are the empty and doubly occupied probabilities. The quantity $2\sqrt{p_{0}p_{2}}$ therefore represents the incoherent background that must be overcome for entanglement to emerge. This mixed-state contribution decreases rapidly with increasing *U*, falling from approximately 0.5 at weak interaction to nearly zero around U=200Γ, while the coherence term $2|\rho_{LR}{|}_{\mathrm{MP}}$ remains roughly constant at 0.02–0.03 over the entire range. Increasing *U* therefore does not primarily generate additional coherence. Instead, it removes the incoherent contribution that masks the existing coherence. The emergence of entanglement is thus controlled by the competition between quantum coherence and Coulomb-induced state purification rather than by the absolute magnitude of coherence alone.

Figure 5c presents the concurrence at the maximum power point. For all dephasing strengths, the concurrence remains zero up to approximately U=180Γ, where the coherence finally exceeds the mixed-state threshold. Beyond this point, the concurrence rises rapidly and reaches about 0.22 in the strong interaction regime, with the coherence-preserving case $\gamma_{\phi }=0$ yielding the largest value and increasing dephasing systematically reducing it. This ordering confirms that entanglement generation requires both strong Coulomb blockade and the preservation of interdot phase coherence. Coulomb interaction alone cannot create entanglement, while coherence alone is insufficient when charge mixing remains strong. Finally, Figure 5d compares the exact concurrence with the transport-based entanglement witness $W_{I,\mathrm{MP}}=2\max\{0,|J_{LR}\left|/(2\right|t_{c}\left|\right)-\sqrt{p_{0}p_{2}}\}$. Since the interdot current JLR=2tcImρLR probes only the imaginary part of the coherence while the concurrence depends on the full magnitude $|\rho_{LR}|$, the current witness is inherently conservative. It becomes nonzero only near U=240Γ, well after the concurrence begins to rise at U=180Γ, and saturates at approximately 0.007, remaining below the exact concurrence of about 0.025. A vanishing current witness therefore does not exclude entanglement, because part of the quantum coherence may reside in the real component of $\rho_{LR}$ invisible to the stationary particle current. Nevertheless, a finite positive $W_{I,\mathrm{MP}}$ provides a direct transport signature of entanglement without requiring full state tomography.

Overall, Figure 5 establishes a complete hierarchy among coherence, Coulomb blockade, and entanglement generation. Strong Coulomb interaction suppresses incoherent charge fluctuations and allows the pre-existing interdot coherence to overcome the separability threshold, producing stationary entanglement. Pure dephasing reduces both coherence and entanglement, while transport measurements offer a practical, albeit conservative, route for detecting a fraction of the generated quantum correlations.

## 5. Conclusions

We have investigated the thermoelectric performance and quantum correlations of a coherent double quantum dot coupled to two ordinary electronic reservoirs. In contrast to schemes that rely on squeezed, bosonic, or otherwise nonthermal environments, the present setup uses only a temperature difference and an electrochemical bias. This minimal configuration isolates the influence of internally generated electronic coherence without introducing an additional energetic resource. The role of coherence was examined by comparing the coherence-preserving dynamics with an energy-conserving pure dephasing channel, ensuring that all observed differences originate from the suppression of the phase relation between the hybridized one-electron states.

The voltage-dependent analysis showed that reducing the dephasing strength enhances the particle current, the absorbed heat current, the output power, and, within the investigated parameter regime, the thermodynamic efficiency. The optimized energy level maps further revealed that coherence modifies mainly the magnitude of thermoelectric performance rather than the location of the favorable operating region. Both the coherent and strongly dephased devices reach their optimal performance when the quantum dot energies lie in the thermally active window, but the coherence-preserving case produces a larger maximum power and a higher efficiency at maximum power. Energy filtering therefore determines where the device operates efficiently, whereas coherence determines how effectively the selected transport channel converts heat into work.

The analysis of quantum correlations revealed a fundamental distinction between coherence and entanglement. A finite interdot coherence is already present at moderate Coulomb interaction, but the concurrence remains zero because the empty and doubly occupied states generate a mixed-state contribution that masks the coherent correlation. Increasing the interdot Coulomb interaction suppresses double occupation and lowers the separability threshold, allowing the pre-existing coherence to overcome it and produce stationary entanglement at the maximum power point. Coulomb blockade therefore acts as a mechanism that converts existing coherence into observable quantum entanglement, not by creating additional coherence but by removing the incoherent background that prevents it from acquiring an entanglement character.

We also constructed a current-based lower bound for the concurrence, which becomes positive only at sufficiently strong interaction because the stationary interdot current captures only the imaginary component of the coherence. Although it detects less entanglement than the full concurrence, a positive current witness certifies stationary entanglement without requiring complete state tomography and provides a direct connection between quantum correlations and experimentally measurable transport quantities.

Taken together, these results establish a clear hierarchy. The quantum dot energies determine the thermoelectric operating window, coherence enhances particle and energy transfer within this window, Coulomb blockade suppresses the incoherent occupation background and enables the surviving coherence to generate entanglement, and transport measurements can reveal a finite part of this entanglement through the current-based witness. The present study therefore demonstrates that a double quantum dot coupled only to conventional electronic reservoirs can simultaneously operate as a finite-power thermoelectric converter and support stationary quantum correlations, providing a route toward identifying and exploiting quantum resources in minimal mesoscopic thermal machines. Future work may extend this framework to current fluctuations, thermodynamic uncertainty relations, finite-frequency noise, and experimentally realistic dephasing mechanisms.

## Data Availability

The original contributions presented in this study are included in the article. Further inquiries can be directed to the corresponding author.

## Appendix A. Explicit Form of the Dissipators

In the eigenbasis {|0〉,|+〉,|−〉,|2〉} the system Hamiltonian is diagonal, $H_{S}=\mathrm{diag}\left(0,E_{+},E_{-},E_{+}+E_{-}+U\right)$. The local annihilation operators are expanded as

(A1) $$\begin{array}{cc} d_{L} & =\cos\varphi \;d_{+}-\sin\varphi \;d_{-},\\ d_{R} & =\sin\varphi \;d_{+}+\cos\varphi \;d_{-}, \end{array}$$

with the mixing angle φ defined in the main text. The operators $d_{\pm }$ annihilate an electron in the hybridized states |+〉 and |−〉, respectively. After decomposing these operators into transition components according to Equation (6) and evaluating the coarse-grained coefficients in the wide-band limit [66], the injection dissipator for the left reservoir is obtained as

(A2) $$\begin{array}{cc} L_{L}^{\mathrm{in}}\rho & =\Gamma_{L}f_{L}\left(E_{+}\right)\left[\cos^{2}\;\varphi \;J\left[d_{+}^{†}\right]\;\rho +\sin^{2}\;\varphi \;J\left[d_{-}^{†}\right]\;\rho -\frac{1}{2}\sin2\varphi \left(J\left[d_{+}^{†},d_{-}\right]\;\rho +J\left[d_{-}^{†},d_{+}\right]\;\rho \right)\right]\\ \; & \;+\Gamma_{L}f_{L}\left(E_{-}\right)\left[\sin^{2}\;\varphi \;J\left[d_{+}^{†}\right]\;\rho +\cos^{2}\;\varphi \;J\left[d_{-}^{†}\right]\;\rho +\frac{1}{2}\sin2\varphi \left(J\left[d_{+}^{†},d_{-}\right]\;\rho +J\left[d_{-}^{†},d_{+}\right]\;\rho \right)\right], \end{array}$$

where we introduced the notation $J\left[A\right]\rho =A\rho A^{†}-\frac{1}{2}\left\{A^{†}A,\rho \right\}$ and $J\left[A,B\right]\rho =A\rho B^{†}-\frac{1}{2}\left\{B^{†}A,\rho \right\}$ for the standard jump and interference terms. The extraction dissipator $L_{L}^{\mathrm{out}}$ is obtained by replacing $f_{L}\to 1-f_{L}$ and $d_{\pm }^{†}\to d_{\pm }$ in Equation (A2). The dissipators for the right reservoir have the same structure with the substitutions φ→φ−π/2 and L→R.

The inclusion of the counting fields amounts to multiplying the jump part of $L_{\nu }^{\mathrm{in}}$ by $e^{i\chi_{\nu }^{N}+i\chi_{\nu }^{E}\bar{\omega }}$ and that of $L_{\nu }^{\mathrm{out}}$ by $e^{-i\chi_{\nu }^{N}-i\chi_{\nu }^{E}\bar{\omega }}$, where $\bar{\omega }$ is the mean transition frequency as defined in the main text. The resulting counting-field-dependent Liouvillian L(χ) is then used to compute the cumulant generating function and all transport quantities.
